# Physical activity and telomere length: Impact of aging and potential mechanisms of action

**DOI:** 10.18632/oncotarget.16726

**Published:** 2017-03-30

**Authors:** Nicole C. Arsenis, Tongjian You, Elisa F. Ogawa, Grant M. Tinsley, Li Zuo

**Affiliations:** ^1^ Department of Nursing, College of Nursing and Health Sciences, University of Massachusetts Boston, Boston, MA, USA; ^2^ Department of Exercise and Health Sciences, College of Nursing and Health Sciences, University of Massachusetts Boston, Boston, MA, USA; ^3^ Department of Kinesiology & Sport Management, Texas Tech University, Lubbock, TX, USA; ^4^ Radiologic Sciences and Respiratory Therapy Division, School of Health and Rehabilitation Sciences, The Ohio State University College of Medicine, Columbus, OH, USA

**Keywords:** telomere length, leukocyte, muscle, physical activity, exercise

## Abstract

Telomeres protect the integrity of information-carrying DNA by serving as caps on the terminal portions of chromosomes. Telomere length decreases with aging, and this contributes to cell senescence. Recent evidence supports that telomere length of leukocytes and skeletal muscle cells may be positively associated with healthy living and inversely correlated with the risk of several age-related diseases, including cancer, cardiovascular disease, obesity, diabetes, chronic pain, and stress. In observational studies, higher levels of physical activity or exercise are related to longer telomere lengths in various populations, and athletes tend to have longer telomere lengths than non-athletes. This relationship is particularly evident in older individuals, suggesting a role of physical activity in combating the typical age-induced decrements in telomere length. To date, a small number of exercise interventions have been executed to examine the potential influence of chronic exercise on telomere length, but these studies have not fully established such relationship. Several potential mechanisms through which physical activity or exercise could affect telomere length are discussed, including changes in telomerase activity, oxidative stress, inflammation, and decreased skeletal muscle satellite cell content. Future research is needed to mechanistically examine the effects of various modalities of exercise on telomere length in middle-aged and older adults, as well as in specific clinical populations.

## INTRODUCTION

Telomeres protect the integrity of information-carrying DNA throughout cell cycle by serving as specialized DNA caps on chromosomes. During successive cellular divisions, telomeres prevent base pair loss of chromosomal DNA. Over time, telomere length decreases until the telomere becomes too short for the cell to divide, resulting in cellular senescence. This scenario is known as the end replication problem and describes the inability of DNA polymerase to replicate the terminal ends of the chromosomes (i.e. the telomeres) [1]. Telomerase, an enzyme with a catalytic unit termed protein reverse transcriptase, serves to combat the end replication problem through lengthening of telomeres [2].

It is suggested that telomere length may be a potential cellular marker for biological aging, as demonstrated by an inverse relationship between age and telomere length [3]. Although telomere length, and its attrition over time, is extremely variable among individuals, it is thought to be stable from childhood through young adulthood but begins to decrease in older adulthood [1]. Moreover, leukocyte telomere length is positively associated with number of years of healthy living, which may indicate that leukocyte telomere length is a biomarker for healthy aging [4].

Current evidence supports the idea that telomere length is associated with a number of chronic conditions including dyslipidemia [5], hypertension [6, 7], atherosclerosis [8, 9], stroke [10], coronary artery disease [11, 12], myocardial infarction [13], and poor cardiovascular disease prognosis [10, 14, 15]. Decreased leukocyte telomere length is also present in patients with colorectal [16, 17] and ovarian cancers [18]. It has been reported to be associated with high risks for gastric cancer [19], early onset of ovarian and breast cancers [20, 21], as well as increased overall cancer incidence and mortality [22]. Furthermore, a number of studies indicate that telomere attrition is positively correlated with diabetes and diabetic complications [23–30]. For example, among type 2 diabetics, those experiencing complications such as diabetic nephropathy have shorter leukocyte telomere lengths than individuals without complications and those without diabetes [31].

It is well known that physical activity is associated with healthy aging and reduced risk for a number of chronic conditions [32], though the relationship between physical activity and telomere length remains unclear. Evidence supports an inverse relationship between telomere length and chronic pain [33, 34] and various psychological stresses [35–39]. Interestingly, in a study that measured stress levels in both sedentary and physically active individuals, perceived stress among sedentary individuals was negatively associated with telomere length, whereas among physically active individuals, perceived stress was not related to telomere length [40]. This suggests that physical activity may confer protection against stress-related telomere length shortening.

Based on the significance of telomere length in aging and the need to understand the potential association with physical activity, the purpose of this systematic review is to investigate whether physical activity and exercise influence telomere length and to discuss possible mechanisms of action.

## SEARCH STRATEGY

A PubMed literature search was performed in accordance with the PRISMA statement [41]. The search focused on leukocyte and skeletal muscle telomere length in relation to exercise or physical activity. Search keywords included: “physical activity” OR “exercise” OR “lifestyle factors” OR “diet” OR “body weight” AND “telomere length” OR “telomerase”, AND “leukocyte” OR “muscle”. Inclusion criteria for each article were: an experimental or observational examination of telomere length in relation to physical activity or exercise at any age, a publication in the English language between 1998 and August 2016, and human participants.

## EFFECTS OF PHYSICAL ACTIVITY AND EXERCISE ON TELOMERE LENGTH

### Observational studies

Observational studies examining the potential relationship between physical activity/exercise and telomere length in skeletal muscle cells and leukocytes are summarized in Table 1 [42–44] and Table 2 [45–62], respectively. These studies were conducted in a broad set of individuals, including men and women, young to older adults, and healthy and chronically ill individuals.

**Table 1:** Effects of physical activity/exercise on skeletal muscle telomere length: observational studies

Study, Year	Participants	Physical Activity/Exercise Type	Influence on Telomere Length
Kadi et al., 2008	14 healthy adults (7 non-lifters, 7 power lifters)	Power lifting; 8±3 years	Longer skeletal muscle telomere length in power lifters *vs*. non-lifters
Rae et al., 2010	37 adults (19 sedentary subjects, 18 endurance runners)	Endurance running; 40 km/week, ≥7 years	Same skeletal muscle telomere length in runners *vs*. sedentary subjects. Shorter telomere length in those with greater number of years training *vs*. fewer number of years training. Shorter telomere length in those with greater number of training hours *vs*. fewer number of training hours.
Osthus et al., 2012	20 young and older men (10 medium activity level, 10 endurance athletes)	Endurance exercise (long distance skiing & track running competitions); Medium activity (moderately physically active)	Longer skeletal muscle telomere length in older athletes *vs*. older medium-activity individuals. Same telomere length in young athletes *vs*. young medium-activity individuals.

**Table 2:** Effects of physical activity/exercise on leukocyte telomere length: observational studies

Study, Year	Participants	Physical Activity/Exercise Type	Influence on Telomere Length
Cherkas et al., 2008	2401 white twin adults	Self-reported physical activity (4 groups based on physical activity levels)	Longer leukocyte telomere length with increasing physical activity level.
Ludlow et al., 2008	69 adults	Various aerobic exercise (divided into quartiles based on exercise energy expenditure: 0-990, 991-2340, 2341-3540, and 93541 kcal/wk)	Longer leukocyte telomere length in 2^nd^ quartile *vs*. 1^st^ and 4^th^ quartile. Same telomere length in 2^nd^ quartile *vs*. 3^rd^ quartile.
Werner et al., 2009	58 young and 46 older adults (47 healthy non-athletes, 57 middle and long-distance runners)	Young athletes: 73±4.8 km/wk Older athletes: 80±7.5 km/wk; 35±2.7 years of training experience	Longer leukocyte telomere length, higher telomere-stabilizing proteins, lower telomere erosion in athletes *vs*. non-athletes. Longer telomere length in older athletes *vs*. older non-athletes. Same telomere length in young athletes *vs*. young non-athletes.
LaRocca et al., 2010	25 healthy young and 32 older adults (30 sedentary subjects, 27 endurance exercisers)	Vigorous aerobic exercise ≥5 days/week, >45 min/day, ≥5 years	Same leukocyte telomere length in older athletes *vs*. older sedentary subjects Same telomere length in young athletes *vs*. young non-athletes.
Song et al., 2010	80 healthy adults	Self-reported physical activity	Self-reported physical activity was not associated with leukocyte telomere length, but was associated with accumulation of DNA damage.
Krauss et al., 2011	944 adults with stable coronary heart disease	Self-reported physical activity	Self-reported physical activity was associated with shorter telomere length, but not after multivariate adjustment
Du et al., 2012	7,813 adult women	Eight possible physical activities, usual walking pace, and the number of flights of stairs climbed daily.	Calisthenics/aerobics-associated increase in leukocyte telomere length (0.10-SD) was observed when comparing the most to the least active women.
Kim et al., 2012	44 healthy postmenopausal women (21 sedentary subjects, 23 habitual exercise participants)	Aerobic and resistance exercise for 60+ minutes, > 3 times per week, for > 12 months	Longer leukocyte telomere length in exercise participants *vs*. sedentary subjects.
Denham et al., 2013	123 males (56 healthy non-marathon runners, 67 ultra-marathon runners)	Ultra-marathon running, average distance 40-100 km/week, ≥2 years	Longer leukocyte telomere length in runners *vs*. non-runners.
Mathur et al., 2013	32 middle-aged adults (15 healthy sedentary subjects, 17 marathon runners)	Marathon running, 32±9 miles/week, 14±11 years	Same leukocyte telomere length in runners *vs*. sedentary subjects.
Borghini et al. 2015	62 adults (20 athletes, 42 sedentary controls)	Endurance training	Longer salivary telomere length in endurance athletes *vs*. sedentary controls.
Loprinzi et al 2015	6503 adults	Movement-based behaviors (moderate-intensity and vigorous intensity physical activity, walking/cycling for transportation, and muscle-strengthening activities)	A clear dose-response relation was observed between movement-based behaviors and leukocyte telomere length.
Soares-Miranda et al 2015	582 older adults	Self-reported physical activity	Cross-sectional and longitudinal analyses showed no significant associations between physical activity and leukocyte telomere length
Saßenroth et al 2015	815 older adults	Self-reported physical activity	Physical activity was positively associated with leukocyte telomere length. Practicing a sport for > 10 years associated with longer telomeres.
Silva et al 2016	46 older adults (15 intensively trained, 16 moderately trained, 15 untrained)	Intensive training: training ≥ 5 days/week (>50 km/week); moderate training: playing volleyball, basketball, or running less than 6 km, 2-3 days/week	Longer leukocyte telomere length in trained *vs*. untrained older adults.
Latifovic et al 2016	477 healthy adults	Self-reported physical activity	More vigorous physical activity was associated with longer leukocyte telomere length.
Loprinzi et al 2016	6474 adults	Self-reported physical activity	Meeting physical activity guidelines for running, but not other modes, was associated with longer leukocyte telomere length.
Kanel et al 2016	203 African and Caucasian school teachers	Objectively measured physical activity	Habitual physical activity was not associated with leukocyte telomere length.

Most of available research indicates that higher physical activity exhibits a positive relationship with longer leukocyte or skeletal muscle telomere length in comparison to a sedentary lifestyle. However, inconsistent results have been reported, and such discrepancies are potentially resulted from different exercise protocols (e.g., duration and intensity of physical activates), measured cell types, and self-reported activities (Table 1 and 2). One informative study reported that subjects in the 1^st^ quartile of exercise energy expenditure (0–990 kcal/wk) and 4^th^ quartile (>3540 kcal/wk) had shorter peripheral blood mononuclear cell (PBMC) telomere length than those in the 2^nd^ quartile (991–2340 kcal/wk). There was no difference in PBMC telomere length between the 2^nd^ and 3^rd^ quartiles, suggesting that moderate levels of physical activity may be beneficial in protecting telomere shortening. Interestingly, telomerase activity in PBMC remains similar in all quartiles of physical activity [46]. This is in contrast with the findings of Werner *et al*. in which they observed that, compared to sedentary adults, active individuals participating in long-distance running showed elevated telomerase activity, increased telomere-stabilizing proteins, down-regulated cell-cycle inhibitors, and decreased telomere erosion in the circulating leukocytes [47]. The conflicting results may be due to different sources of telomerase (PBMC *vs*. leukocytes) and a discrepancy in sample sizes between the two studies (69 *vs*.104 subjects). More recent studies support that there is a clear dose-response relationship between movement-based behaviors and telomere length. Accordingly, greater vigorous physical activity is associated with a longer telomere length, as assessed in leukocytes and salivary DNA (Table 2).

Three independent studies evaluated the effect of moderate to vigorous aerobic exercise on skeletal muscle or leukocyte telomere length in young and older adults [44, 47, 48]. In elderly population, athletes had significantly longer skeletal muscle or leukocyte telomeres than non-athletes. However, athletic status appeared to show no marked association with telomere length in young adults. It is likely that telomere lengths in sedentary young adults have not yet experience attrition, but may eventually being affected by reduced telomerase activity if the sedentary lifestyle continues (Table 1 and 2).

Several researchers investigated additional factors that may correlate with telomere length. Studies showing differences between older athletes and non-athletes in their skeletal muscle or leukocyte telomere length also reported positive correlations between telomere length and aerobic fitness (VO_2_ max) [46, 47]. In another study comparing marathon runners and sedentary adults, no difference in leukocyte telomere length was observed between the two groups, nor was there a correlation between telomere length and VO_2_ max across groups [54]. This lack of association may be due to study design considerations, such as the small sample size and a large variation in subjects’ marathon running histories; alternatively, the high activity of marathon runners may also have detrimental effects on telomeres as mentioned previously. Among athletes, leukocyte telomere length has not been shown to be associated with body mass index (BMI) [53]. However, in healthy non-active subjects, there is an inverse correlation between leukocyte telomere length and BMI [45, 49].

The evidence indicates that individuals engaged in multiple healthy behaviors are more likely to maintain telomere length. Telomere length is likely altered by a variety of factors, and some may not yet be known, which could explain the mixed findings between physical activity/exercise and telomere length. For instance, a recent study examining a nationally representative US population showed that leukocyte telomere length moderately correlates with known cardiovascular risk factors such as blood glucose levels, thus serving as a potential predictor of cardiovascular diseases. Specifically, the percentage of body fat demonstrated a strong negative correlation with leukocyte telomere length regardless of age and health behaviors (e.g., physical activities) [63]. Psychological variables were also concurrently associated with telomere length as high anxiety and defensiveness are related to longer and shorter leukocyte telomeres, respectively [64]. Other factors, such as nutritional intake and its relation to oxidative stress and telomere length, are discussed in subsequent sections of this review. To date, many of the presently examined studies have been cross-sectional and involved small sample sizes, often comparing extremes of physical activity. However, even data from large observational studies examining a wide range of physical activity levels indicate that the association between physical activity and telomere length is not entirely clear. Weischer *et al*. examined relative leukocyte telomere length in 4,576 individuals on two occasions, with 10 years between each measurement [65]. The results of this analysis indicated that physical inactivity was associated with short telomere length, but not with the change in telomere length over the course of the 10-year observation period. These data, from a large pool of healthy participants, cast doubt on the influence of physical activity on telomere length [65].

### Interventional studies

There are three reports of randomized controlled intervention study that investigated the effects of exercise on leukocyte telomere length, as summarized in Table 3 [66–68]. A study performed by Mason *et al*. showed that leukocyte telomere length is positively associated with baseline VO_2_ max and inversely associated with age, while unassociated with BMI and percent body fat [67]. However, no significant change in telomere length was found after exercise interventions, which is in accordance with the results of other two studies (Table 3). Despite the lack of an exercise effect on leukocyte telomere length, Sjögren *et al*. reported a strong negative correlation between telomere length and sitting time in the exercise training group but not in the sedentary control group, suggesting a greater importance of reduced sitting time than increased exercise time in telomere maintenance [68].

**Table 3:** Effects of exercise training on leukocyte telomere length: interventional studies

Study, year	Participants	Intervention	Effects on telomere length
Shin et al., 2008	16 obese, middle-aged women (8 control, 8 exercise)	No exercise control *vs*. aerobic exercise training, 6 months	Same leukocyte telomere length in aerobic exercise *vs*. control.
Mason et al., 2013	439 overweight, obese, older adult (87 control, 188 dietary weight loss, 117 aerobic exercise, 117 diet & exercise)	No exercise control *vs*. aerobic exercise training, 5 days per week, 12 months	Same leukocyte telomere length in aerobic exercise versus control.
Sjögren et al., 2014	49 overweight and abdominally obese older adults	Usual care with minimal intervention control *vs*. moderate-intensity aerobic/strength/flexibility/ balance exercise intervention, most days of the week for 6 months	Same leukocyte telomere length in exercise intervention *vs*. control. Longer telomere length with decreased sitting time in exercise group.

In a small quasi-experiment study, investigators studied the effects of acute exercise on leukocyte recruitment and subsequent mean telomere length in young and old subjects [69]. After a single maximal bicycle exercise session, the mean telomere length in selected leukocyte subsets was shorter, likely due to recruitment of cells with a greater history of replication. These findings demonstrate that acute periods of exercise can lead to lower mean estimates of telomere length. Thus, the authors concluded that measures of leukocyte telomeres should not be obtained immediately following vigorous activity. Another acute exercise study observed that immediately after an episode of acute exercise, telomere length of CD8+ T-cells increased, whereas those of CD4+ and CD3+ cells did not. In addition, terminally differentiated CD8+ T-cells were released into the blood, which may indicate that more frequent exercise may allow naïve T cells to populate and improve immune function [70]. It has also been reported that an acute exposure of ultra-distance endurance trail race reduced salivary telomere length in athletes, yet chronic endurance training provided protections against such shortening [55].

## MECHANISMS ASSOCIATED WITH TELOMERE SHORTENING

**Figure 1:**
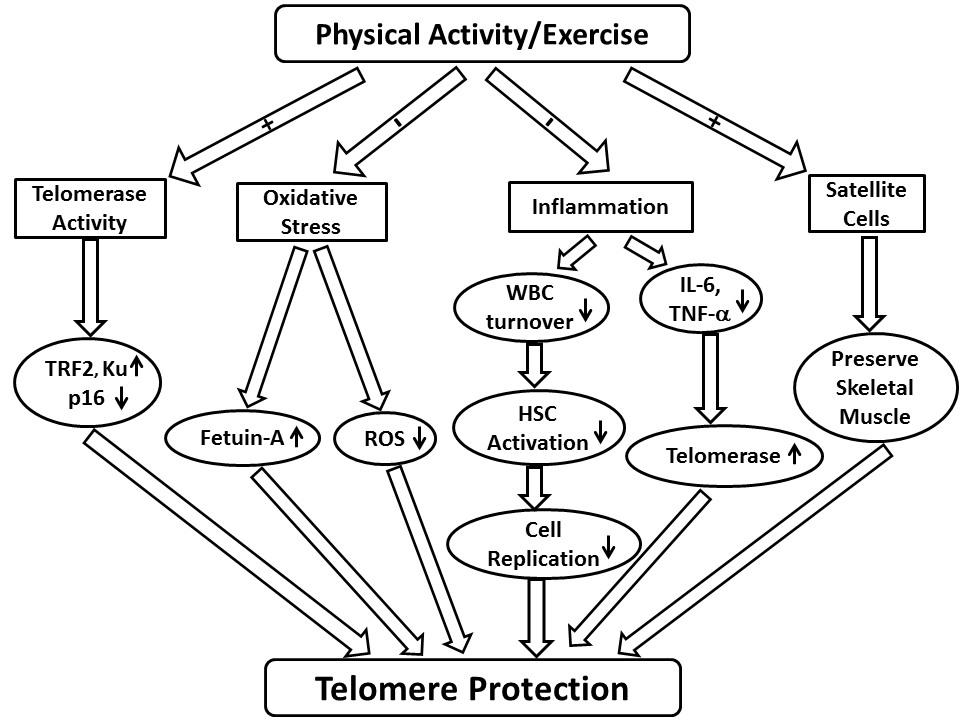
Schematic demonstrating the potential effects of physical activity and exercise on telomere length Abbreviations: TRF2 (telomeric repeat-binding factor 2); ROS (reactive oxygen species); WBC (white blood cell); HSC (haematopoietic stem cell); IL (interleukin); TNF-α (tumor necrosis factor-α).

Both chronic diseases and the aging process are known to be associated with decreased telomere length, but it may be possible to preserve telomere length with physical exercise. Although the existing literature does not provide adequate experimental evidence to fully establish the mechanistic relationship between physical exercise and telomere length, several potential mechanisms exist to explain how physical activity may affect telomere length: telomerase activity, oxidative stress, inflammation, and skeletal muscle satellite cell content (Figure 1).

### Telomerase activity

While telomere length shows a progressive, age-related shortening, telomerase demonstrated a biphasic pattern of expression. Iwama *et al*. reported that from age of 4 to 39 years, there is a progressive decrease in both telomere length and telomerase activity. However, in individuals aged 40 or over, where telomere length continued to progressively decrease, 65% of individuals had a stable but low telomerase activity, and the remaining 35% of individuals had no telomerase activity [71]. Physical activity could play a role in these processes. Werner *et al*. compared track and field athletes to sedentary adults, and found an up-regulation of telomeric repeat-binding factor 2 (TRF2), a protein that plays a role in protecting telomeres from shortening, in the athletes (both young and middle-aged) [47]. In young athletes, there was no change in the expression of p16, a negative regulator of cell cycle progression, nor Ku proteins, which are part of the DNA repair pathway. However, in middle-aged athletes, there was a down-regulation in p16 and up-regulation in Ku mRNA. These results suggest that paralleled with the regulation of TRF2, other transformation associated proteins such as p16 and Ku proteins may play a role in telomere protection in response to exercise for middle-aged individuals. No difference in leukocyte telomere length was observed between young athletes and young sedentary individuals. However, middle-aged sedentary adults had shorter telomeres compared to middle-aged athletes (Table 2). This agrees with the observed up-regulation of TRF2 in young and middle-aged athletes, as well as the observed upregulation of Ku proteins in middle-aged athletes.

### Oxidative Stress

Excessive reactive oxygen species (ROS) production can cause oxidative stress in cells, tissues or organs [72–74], leading to DNA damage and senescence or apoptosis [75]. Starr *et al*. determined that several oxidative-stress genes are linked with both telomere attrition and biological aging [76]. Moreover, decreased fetuin-A levels, a negative acute phase protein and mediator of redox homeostasis, have been associated with shortened leukocyte telomeres and increased risk for colorectal cancer and other diseases [16]. Correlations between shorter leukocyte telomeres and increased oxidative stress have also been reported in diabetics [24, 28]. Furthermore, telomere length was inversely associated with oxidative stress among the Framingham Heart Study Cohort in hypertensive individuals [77]. In fact, telomere length has been put forth as a potential marker of chronic oxidative stress because constant oxidative stress can compromise the repair mechanisms of telomeric DNA [78]. It has been proposed that chronic exercise may lower oxidative stress and therefore protect telomeres from shortening inflicted by excessive ROS [79]. Although oxidative stress can be detrimental to telomeres, proper levels of ROS may be protective. For example, a recent study reported that sperm telomere is lengthened by mild oxidative stress, although it is shortened by severe oxidative stress [80]. Therefore, it appears that the relationship between telomere length and oxidative stress is delicately balanced, whereby certain amount of ROS can aid in telomere maintenance, but this level must be lower than some thresholds to be less detrimental. Lifestyle factors other than exercise potentially play an important role in the modulation of telomere length through altering redox status. A recent research update by Freitas-Simoes and colleagues summarized the current body of evidence concerning dietary factors that impact telomere length, through oxidation and other mechanisms [81]. Diets that are higher in antioxidants from vegetables and whole grains appear to enhance telomere maintenance, and omega-3 fatty acids have emerged as a promising-but-understudied strategy to combat telomere attrition over time [82].

### Inflammation

The attenuation of chronic inflammation provides another potential mechanism for the protective effects of exercise and physical activity on telomeres. For instance, chronic systemic inflammation elevates white blood cell (WBC) turnover, which increases telomere attrition rate [83]. With increased WBC turnover, the division of haematopoietic stem cells is activated, increasing cellular replication and subsequently leading to telomere shortening. Also, the pro-inflammatory cytokine tumor necrosis factor (TNF)-α may cause telomere shortening by downregulating telomerase [83]. Shortened leukocyte telomeres can be linked with elevated concentrations of both interleukin (IL)-6 and TNF-α. In addition, individuals with elevated concentrations of both IL-6 and TNF-α were more likely to have shortened leukocyte telomeres than those with high concentrations of only one of these molecules [84]. Of note, C-reactive protein (CRP) was not associated with telomere length in the same study, though others have observed a negative relationship between telomere length and CRP levels among patients with type-2 diabetes [85].

Chronic obstructive pulmonary disease (COPD) patients have been reported to possess shorter mean leukocyte telomere length than healthy controls, which may be linked with chronic inflammation status in these patients. Specifically, IL-6 levels were inversely correlated with telomere length in COPD [86]. Zhang *et al*. recently described the complex interrelationship between aging, inflammation, and telomere length [87]. They draw attention to a number of proteins that may be involved in the induction of inflammation and telomere dysfunction, such as nuclear factor kappa B, Poly (ADP-ribose) polymerase 1, Wnt, repressor-activator protein 1, and telomerase reverse transcriptase. The manipulation of these proteins as a therapeutic strategy holds promise for combating inflammation and aging-related diseases. Physical activity is negatively related to levels of circulating inflammatory markers, and exercise training contributed to reduced levels of inflammatory markers in individuals with enhanced chronic inflammation [88, 89].

### Decreased satellite cells

Satellite cells are skeletal muscle cell precursors which can regenerate muscle cells or additional satellite cells in response to muscle injury. After age 70, the number of satellite cells is reported to decrease [90]. This could contribute to the reductions of muscle mass seen in sedentary individuals, particularly during aging. It is estimated that approximately 40% of muscle tissue is lost by age 70 in sedentary individuals [91]. In older women, a positive correlation between the quantity of satellite cells and skeletal muscle telomere length has been reported [47], and satellite cells may serve as a mechanism through which physical activity can preserve skeletal muscle in older adults [90]. Physical activity acts to stimulate the satellite cell pool, which counteracts the decline of satellite cells that occurs with aging. Thus, satellite cell content represents another modifiable factor that can be altered in response to different physical activity levels and is therefore a potential player in the association between telomere length and physical activity. It has been suggested that the shortening of satellite cell telomeres leads to a diminished replication capacity in satellite cells [92]. This mechanism may be important in explaining age-related loss of muscle mass.

## CLINICAL SIGNIFICANCE AND IMPLICATIONS

Recent observational and experimental studies reveal a possible relationship between physical activity/exercise and telomere length, although this is not entirely clear from the present body of literature. Evidence has shown that physical activity decreases chronic inflammation and oxidative stress, especially in older, obese individuals, and may thus reduce telomere shortening observed with aging [67]. Therefore, exercise may potentially have a protective effect on telomere length, and may be especially beneficial for those at risk for or living with chronic conditions with high oxidative stress and inflammation.

In addition to physical activity, weight loss itself may reverse telomere attrition. Since age 50, women who were overweight or obese and later lost weight to be normal, or women who sustained a constant weight within 5%, has shown greater leukocyte telomere length than those who gained weight after age 50 [93]. By maintaining a healthy weight and practicing healthy living through physical activity and dietary measures, individuals may be able to maintain an adequate telomere length and protect themselves from harmful effects of excessive inflammation and oxidative stress. For individuals with compromised musculoskeletal function, exercise may have therapeutic, preventive, and rehabilitative effects. Both resistance and aerobic trainings have been shown to increase the number of satellite cells, which may be important for regulating skeletal muscle telomere length [90]. Future studies should elucidate mechanisms of variations in telomere length, especially in adipose tissue, which may particularly provide more insight into the telomere biology in obese persons.

## CONCLUSIONS

Telomere length has inverse associations with chronic conditions (including cardiovascular disease, obesity, and diabetes). Physical activity and exercise may be beneficial for telomere length maintenance in both healthy and chronically ill middle-aged and older adults. Telomere length is not only a marker of aging, but also relates to the ability to protect DNA from damage and the associated consequences. Individuals living with chronic conditions are more likely to be sedentary and experience functional limitations and disability. Physical activity and exercise may have both protective and restorative effects, and as such, show great potential to improve well-being and increase longevity. However, more interventional studies, especially those with longer-term exercise, are needed to confirm specific effects of various doses and intensities of exercise training on telomere length, particularly in middle-aged and older adults who are at increased risk for chronic diseases associated with inflammation and oxidative stress.
